# Cluster habitat-based diffusion MRI radiomics for differentiation of glioblastoma from solitary brain metastasis

**DOI:** 10.1186/s41747-026-00717-y

**Published:** 2026-04-27

**Authors:** Wenzheng Luo, Yuanhao Cheng, Changhe Pang, Shenghui Xie, Zhiyue Hao, Yanhao Liu, Mingxue Hu, Xiaoyue Ma, Shanshan Zhao, Mengzhu Wang, Yang Song, Chengxiu Zhang, Yong Zhang, Yang Gao, Guohua Zhao

**Affiliations:** 1 Department of Neurosurgery, First Affiliated Hospital of Zhengzhou University, Zhengzhou, China; 2 School of Intelligent Engineering, Xi’an Jiaotong Liverpool University, Suzhou, China; 3 Department of Radiology, Affiliated Hospital of Inner Mongolia Medical University, Hohhot, China; 4 Ordos Clinical Medical College, Inner Mongolia Medical University, Hohhot, China; 5 Department of Magnetic Resonance Imaging, First Affiliated Hospital of Zhengzhou University, Zhengzhou, China; 6 MR Research Collaboration, Siemens Healthineers Ltd., Shanghai, China; 7 Shanghai Key Laboratory of Magnetic Resonance, East China Normal University, Shanghai, China

**Keywords:** Brain neoplasms, Cluster analysis, Glioblastoma, Magnetic resonance imaging, Radiomics

## Abstract

**Objective:**

Given the distinct intratumoral heterogeneity of glioblastoma (GB) and solitary brain metastasis (SBM), habitat-based radiomics derived from neurite orientation dispersion and density imaging (NODDI) may offer enhanced diagnostic value. This study aimed to evaluate NODDI habitat analysis in distinguishing GB from SBM.

**Materials and methods:**

This retrospective, two-center study included 279 patients (196 GB, 83 SBM) who underwent 3-T magnetic resonance imaging (MRI), including T1-, T2-, and diffusion-weighted as well as contrast-enhanced T1-weighted sequences. K-means clustering was performed on NODDI images within the region of interest. Following feature extraction, five models were developed: habitat subregion, habitat, radiomics, clinical, and combined. Performance was evaluated using the area under the receiver operating characteristic curve (AUROC), calibration curves, and decision curve analysis.

**Results:**

The region of interest was divided into three habitat subregions, with the Habitat-H2 subregion demonstrating strong discriminatory ability (validation AUROC = 0.900; testing AUROC = 0.828). Compared to the radiomics and clinical models, the habitat model containing the three subregions showed a higher discriminatory ability (validation AUROC = 0.929; testing AUROC = 0.851). The combined model, integrating habitat features and clinical variables (age) into a nomogram, achieved the highest predictive performance (validation AUROC = 0.931; testing AUROC = 0.912) and provided superior clinical value.

**Conclusion:**

NODDI-based habitat MRI radiomics shows potential for differentiating GB from SBM, while integrating clinical variables is essential for optimal diagnostic performance.

**Relevance statement:**

NODDI-based habitat radiomics aids differentiation between glioblastoma and solitary brain metastasis.

**Key Points:**

- Habitat analysis based on NODDI facilitates the differentiation between GB and SBM on MRI.
- The benefits of habitat analysis are maximized in the three clustered subregions.
- Habitat radiomics captures intratumoral heterogeneity that whole-tumor radiomics may miss.

**Graphical Abstract:**

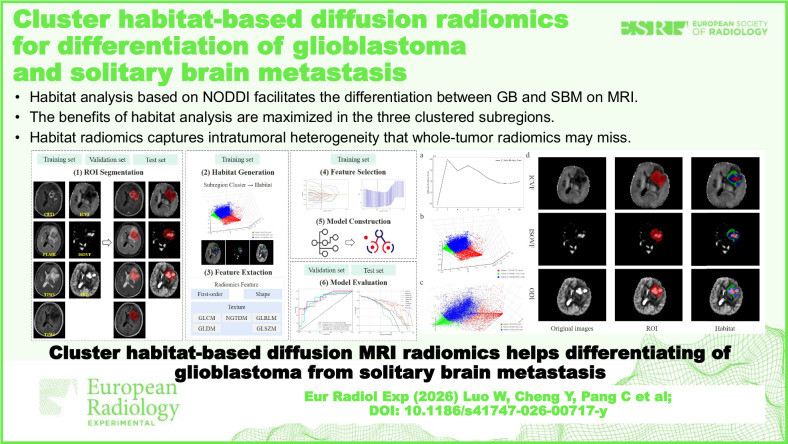

## Background

Glioblastoma (GB) and solitary brain metastasis (SBM) are the most common malignant brain tumors in adults, with significant differences in their clinical manifestations, pathological features, and treatment strategies [1, 2]. GB accounts for more than half of all primary central nervous system malignancies, is highly invasive, and has a poor prognosis [3]. SBM results from hematogenous metastases of malignant tumors originating from other parts of the body, and its treatment and prognosis depend on the primary lesion [4]. Approximately 30% of patients with brain metastases present with brain lesions as the initial manifestation, and nearly half of these cases involve SBM. Accurately distinguishing between GB and SBM is crucial for developing personalized treatment plans. Although histopathology is the gold standard for diagnosis, biopsy or surgery carries a higher risk in older or frail patients. Magnetic resonance imaging (MRI) is the preferred imaging modality for diagnosing GB and SBM; however, conventional MRI alone often cannot reliably distinguish GB from SBM [5, 6]. Therefore, the development of safe and accurate non-invasive imaging methods has important clinical significance.

Recent studies have indicated that diffusion imaging, particularly advanced neurite orientation dispersion and density imaging (NODDI), has considerable potential for differentiating GB from SBM [7–12]. NODDI, a multi-shell spherical diffusion model, exploits differences in the diffusion of water molecules inside and outside cells, thereby enabling quantitative evaluation of brain microstructural complexity and heterogeneity [6, 7]. Multiple studies have examined the utility of NODDI in distinguishing GB from SBM using both quantitative and radiomics analyses. Quantitative analysis supports differentiation by comparing statistical variations in pixel values within regions of interest (ROIs) [8–10]. However, such analyses rely on sparse quantitative features that often fail to elucidate the intricate biological characteristics underlying these signals, thereby limiting their application in exploratory research. In contrast, radiomics analysis can automatically extract numerous quantitative features from images and distinguish between GB and SBM by constructing predictive models [11–13]. Nevertheless, radiomics features predominantly reflect the global quantitative attributes of a given ROI and are insufficient for characterizing the spatial partitioning and heterogeneity within the ROI.

Habitat imaging partitions tumor regions into distinct habitat subregions, each characterized by unique biological properties, thereby reflecting spatial heterogeneity and microenvironmental diversity within the tumor [14–18]. Several studies have indicated that different tumor habitats may exhibit unique growth patterns and invasion, resulting in the regional heterogeneity of tumor genotypes and phenotypes. Radiomics features extracted from intratumoral habitats have demonstrated practical value in a variety of applications, including prognostic prediction for IDH wild-type GB and spatiotemporal imaging evaluation of the local recurrence of high-grade glioma [16, 17]. For tumors exhibiting markedly different degrees of heterogeneity, such as GB and SBM, habitat-based radiomics may provide more pronounced advantages for differential diagnosis.

In this study, we evaluated the utility of NODDI-based habitat radiomics analysis for distinguishing GB from SBM, compared its discriminative performance with clinical and radiomics models, and interpreted the biological underpinnings of the NODDI habitat radiomics models.

## Methods

This retrospective study was approved by the institutional review boards (IRBs) of the two participating institutions, both of which waived the requirement for written informed consent from patients. The study was conducted in accordance with the principles of the Declaration of Helsinki.

### Patients

**Fig. 1:**
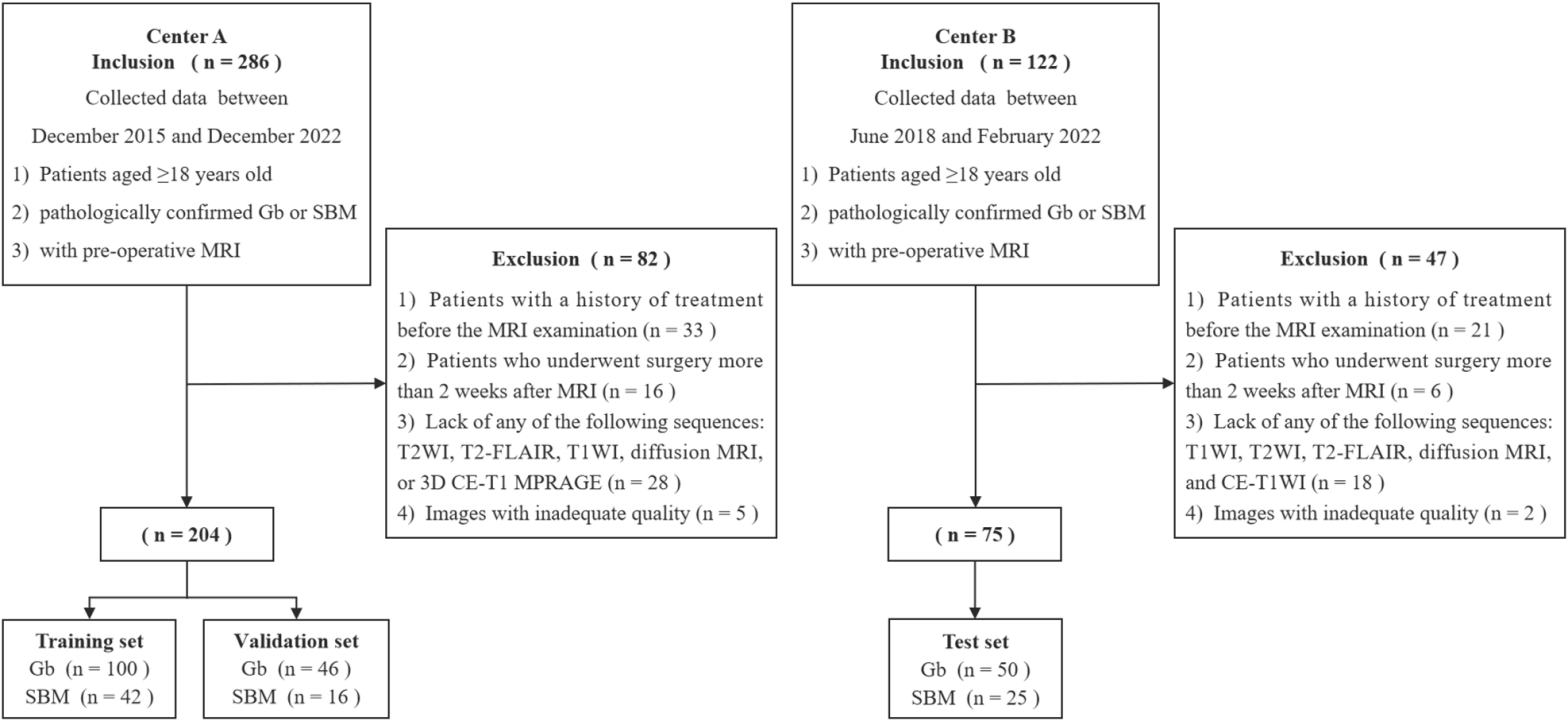
Flow diagram of the patient inclusion and exclusion process

**Fig. 2:**
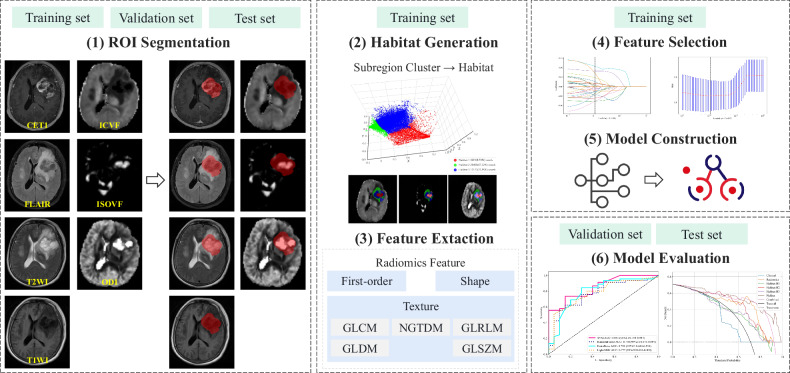
The overall workflow of this study

Patients were recruited from two medical centers. This retrospective study screened the medical records of patients with histologically confirmed GB or SBM from Center A (December 2015 to December 2022) and Center B (June 2018 to February 2022) according to the inclusion and exclusion criteria, which are shown in Fig. 1. All enrolled patients were classified according to the 2016 World Health Organization guidelines. A total of 204 patients from Center A fulfilled the research criteria and were randomly assigned to training and internal validation sets in a 7:3 ratio. Additionally, 75 patients from Center B who met the study criteria were included in the external test set. Demographic and clinical characteristics are summarized in Table 1. The study design and workflow are illustrated in Fig. 2.

**Table 1:** Clinical characteristics of patients whose data were included in the training and test datasets

Characteristic	Training dataset (*n* = 142)			Validation dataset (*n* = 62)			Test dataset (*n* = 75)		
	GB (*n* = 100)	SBM (*n* = 42)	*p*-value	GB (*n* = 46)	SBM (*n* = 16)	*p*-value	GB (*n* = 50)	SBM (*n* = 25)	*p*-value
Age, years									
Mean ± standard deviation	53.2 ± 11.2	57.1 ± 10.2	0.053	53.1 ± 9.9	56.4 ± 10.9	0.266	52.7 ± 9.5	56.8 ± 11.2	0.101
Sex, *n*									
Male (%)	58 (58.0)	24 (57.1)	0.926	25 (54.3)	10 (62.5)	0.570	28 (56.0)	15 (60.0)	0.740
Female (%)	42 (42.0)	18 (42.9)		21 (45.7)	6 (37.5)		22 (44.0)	10 (40.0)	
Variety of SBM, *n*									
Lung, *n*									
Adenocarcinoma (%)		29 (69.0)			11 (68.8)			18 (72.0)	
Squamous cell carcinoma(%)		2 (4.8)							
Neuroendocrine carcinoma (%)		3 (7.1)			2 (12.6)			2 (8.0)	
Small cell lung carcinoma (%)		1 (2.4)						1 (4.0)	
Poorly differentiated carcinoma (%)		1 (2.4)						1 (4.0)	
Stomach, *n*									
Adenocarcinoma (%)		1 (2.4)						1 (4.0)	
Kidney, *n*									
Clear cell carcinoma (%)		3 (7.1)			1 (6.2)			1 (4.0)	
Uterus, *n*									
Endometrial carcinoma (%)		1 (2.4)			1 (6.2)			1 (4.0)	
Unknown site, *n* (%)		1 (2.4)			1 (6.2)				

*GB* Glioblastoma, *SBM* Solitary brain metastasis

### MRI acquisition and image processing

3-T MRI scanners (MAGNETOM Prisma and MAGNETOM Skyra; Siemens Healthineers) were used to perform the MRI examinations. Comprehensive details regarding the multicenter scanning equipment, imaging parameters, and image processing methods are provided in Supplementary Material E1 and Supplementary Tables S1 and S2. The MR data included conventional MRI sequences: T1-weighted images, T2-weighted images, fluid-attenuated inversion recovery-FLAIR (fluid-attenuated inversion recovery), three-dimensional contrast-enhanced T1 magnetization-prepared rapid gradient echo (Center A), contrast-enhanced T1-weighted images (Center B), and diffusion-weighted images. NODDI parameter maps, including isotropic volume fraction (ISOVF), intracellular volume fraction (ICVF), and orientation dispersion index (ODI), were calculated from diffusion MRI data using in-house post-processing software, NeuDiLab, developed based on the open-source DIPY Toolbox (http://dipy.org). All imaging volumes were resampled to an isotropic voxel size of 1 × 1 × 1 mm^3^ to ensure spatial uniformity.

### Habitat imaging

Habitat imaging analysis divides a given ROI into subregions. In this study, the ROI, representing the solid tumor, was delineated using an automatic segmentation algorithm [19]. Details of the automatic segmentation process are provided in Supplementary Material E2.

To delineate intratumoral subregions in GB and SBM, a data-driven K-means clustering algorithm was employed using voxel-wise signal intensities as input features. For each tumor ROI, voxel values from the ICVF, ISOVF, and ODI parameter maps were concatenated to construct multimodal feature vectors. These feature vectors were initially extracted from all voxels within the ROI for each subject and subsequently aggregated across the entire cohort, resulting in a global feature matrix in which rows represented individual voxels and columns corresponded to the three imaging parameters.

This global feature matrix was then subjected to K-means clustering to enable the unsupervised segmentation of the tumor into multiple subregions characterized by homogeneous signal profiles across the selected parameters. To optimize the granularity of segmentation, the number of clusters was systematically varied from 2 to 10, and the optimal cluster number was determined by maximizing the Calinski–Harabasz score. All clustering analyses and performance evaluations were conducted using the scikit-learn library in Python.

### Feature extraction

Feature extraction was performed independently for each habitat subregion across all three imaging modalities. A total of 1,547 handcrafted radiomic features were extracted from each habitat subregion, including 306 first-order statistical features, 14 shape features, and a set of texture features derived from the gray-level co-occurrence matrix‒GLCM, gray-level run-length matrix‒GLRLM, gray-level size zone matrix‒GLSZM, neighborhood gray-tone difference matrix‒NGTDM, and gray-level dependence matrix‒GLDM. All features were extracted using the PyRadiomics library (version 3.0.1) following the guidelines of the Imaging Biomarker Standardization Initiative‒IBSI to ensure reproducibility and methodological consistency. To address fragmented or unlabeled voxels within the clusters, the k-nearest neighbor (KNN) algorithm was employed to propagate labels based on neighborhood consistency. Subsequently, a prefusion strategy was adopted to aggregate the features from individual subregions into a comprehensive set of habitat features, thereby enhancing the discriminative power of the downstream predictive modeling.

### Feature selection

A multistage feature selection strategy was employed to reduce dimensionality while preserving essential information. Initially, univariate statistical tests (*p* < 0.05) were used to identify significant features. Redundant variables were then removed using Pearson’s correlation analysis (|r| > 0.9) to mitigate multicollinearity. The minimum redundancy maximum relevance‒mRMR algorithm was applied to rank the remaining features, and the top 32 were selected for model development. Subsequently, the least absolute shrinkage and selection operator (LASSO) regression was applied to induce sparsity, with the optimal regularization parameter (λ) determined *via* 10-fold cross-validation to enhance model generalizability and interpretability.

### Model construction

Linear classifiers such as support vector machines (SVM) and ensemble-based algorithms (including random forests, extra trees, and light gradient boosting machine) were used to capture linear and nonlinear features in model building. The habitat model was constructed by integrating radiomics features derived from spatially clustered tumor subregions, thereby modeling intratumoral heterogeneity. Furthermore, predictive models were developed independently for each habitat subregion (denoted as Habitat-Hx, where “x” represents the subregion index) to facilitate a comparative analysis of their predictive capabilities. For comparison, radiomics and clinical models were constructed. The radiomics model was developed using features extracted from the entire ROI. The clinical model included only sex and age as available features, which were directly modeled using the classifier. An integrated combined model was also established using multivariate logistic regression, incorporating clinical and habitat features. Only features with univariate significance (*p* < 0.05) were retained to enhance model parsimony and interpretability. Model performance was systematically evaluated on the internal validation and external test sets using: (1) receiver operating characteristic‒ROC analysis and area under the ROC curve (AUROC) for discrimination; (2) calibration plots with the Hosmer–Lemeshow test for calibration; and (3) decision curve analysis (DCA) to assess clinical utility across varying decision thresholds.

### Statistical analysis

Clinical variable distributions were evaluated using the Shapiro–Wilk test. Continuous variables were compared using the Student *t*-test or Mann–Whitney *U* test, contingent upon their distribution, and categorical variables were analyzed using the chi-squared test. All analyses were conducted on the OnekeyAI platform (v3.5.12) with Python 3.7.12 and Statsmodels 0.13.2. Feature extraction was performed using PyRadiomics (v3.0.1), and machine learning models, including SVM, were implemented using Scikit-learn (v1.0.2).

## Results

### Patient characteristics

The clinical characteristics of each patient group are summarized in Table 1. No significant differences in clinical characteristics were observed among the training, validation, and test sets (all *p* > 0.05). Across the two centers, 196 patients with GB and 83 patients with SBM were included. The proportions of GB in the training and validation sets were 70.4% (100/142) and 74.2% (46/62), respectively, without a significant difference between the two sets (*p* = 0.571).

### Habitat imaging and feature selection

**Fig. 3:**
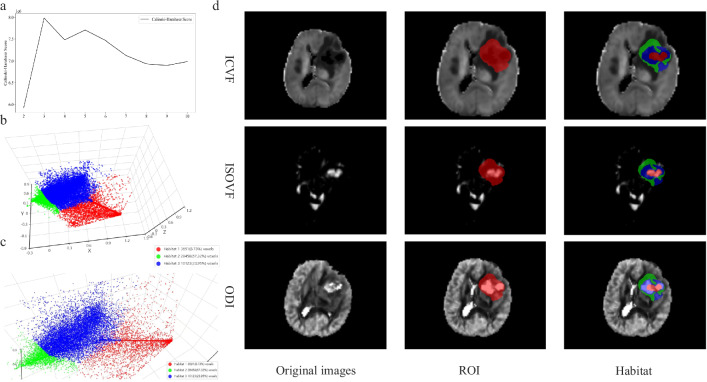
Visualization of habitat clustering. Calinski–Harabasz score of different clusters (**a**), three-dimensional spatial projection of each subregion’s features from different perspectives (**b**, **c**), and habitat regions generated (**d**)

To determine the optimal number of tumor subregions, we evaluated cluster counts ranging from 2 to 10 using the Calinski–Harabasz score, which was observed at three clusters, indicating that this configuration provided the best balance between intercluster separation and intracluster compactness. Figure 3 shows the Calinski–Harabasz scores, a three-dimensional representation of the resulting clustered subregions, and an example of the generated habitat image.

For feature selection, we employed the least absolute shrinkage and selection operator (LASSO) logistic regression model to identify non-zero coefficient features that are critical for constructing the radiomics score. Supplementary Figs. S1‒S5 illustrate the feature selection process for the habitat model, habitat subregion (Habitat-H1, Habitat-H2, and Habitat-H3) models, and radiomics models.

### Performance of different models

**Fig. 4:**
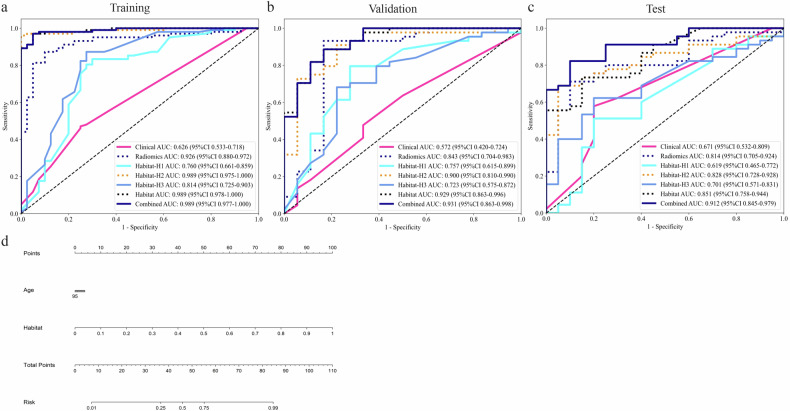
Receiver operating characteristic curves (**a**–**c**) for different models and nomograms (**d**) for combined models

Among the individual habitat subregions, Habitat-H2 exhibited the highest predictive performance across all datasets. Specifically, in the validation set, H2 yielded an AUROC of 0.900, exceeding those of H1 (0.757) and H3 (0.723); similarly, in the test set, H2 maintained its leading performance with an AUROC of 0.828, outperforming H1 (0.619) and H3 (0.701). The H2 subregion displayed the most robust predictive capability, suggesting that it may constitute a biologically informative zone contributing most significantly to classification performance. The habitat model, constructed by fusing the image features of the three habitat subregions, achieved AUROCs of 0.929 and 0.851 for the validation and test sets, respectively. These results indicated that the habitat model outperformed the three habitat subregion models. Table 2 lists the performance metrics of all models, and Fig. 4 shows the ROC curves. Supplementary Tables S3‒S8 detail model performance under different classifiers.

**Table 2:** Performance of the different models

Model name	Accuracy	AUROC	95% CI	Sensitivity	Specificity	PPV	NPV	Dataset
Clinical	0.549	0.626	0.533–0.718	0.471	0.750	0.828	0.357	Training
Radiomics	0.880	0.926	0.879–0.972	0.873	0.900	0.957	0.735	Training
Habitat-H1	0.796	0.760	0.661–0.858	0.833	0.700	0.876	0.622	Training
Habitat-H2	0.972	0.989	0.974–1.000	0.961	1.000	1.000	0.909	Training
Habitat-H3	0.831	0.814	0.725–0.903	0.873	0.725	0.890	0.690	Training
Habitat	0.965	0.989	0.978–1.000	0.971	0.950	0.980	0.927	Training
Combined	0.965	0.989	0.977–1.000	0.971	0.950	0.980	0.927	Training
Clinical	0.532	0.572	0.420–0.723	0.477	0.667	0.778	0.343	Validation
Radiomics	0.903	0.843	0.703–0.983	0.932	0.833	0.932	0.833	Validation
Habitat-H1	0.774	0.757	0.614–0.899	0.795	0.722	0.875	0.591	Validation
Habitat-H2	0.871	0.900	0.810–0.990	0.909	0.778	0.909	0.778	Validation
Habitat-H3	0.710	0.723	0.574–0.872	0.682	0.778	0.882	0.500	Validation
Habitat	0.871	0.929	0.862–0.995	0.886	0.833	0.929	0.750	Validation
Combined	0.871	0.931	0.863–0.998	0.886	0.833	0.929	0.750	Validation
Clinical	0.653	0.671	0.531–0.809	0.580	0.800	0.853	0.488	Test
Radiomics	0.773	0.814	0.705–0.923	0.700	0.920	0.946	0.605	Test
Habitat-H1	0.600	0.619	0.465–0.772	0.500	0.800	0.833	0.444	Test
Habitat-H2	0.773	0.828	0.727–0.927	0.680	0.960	0.971	0.600	Test
Habitat-H3	0.680	0.701	0.571–0.831	0.620	0.800	0.861	0.513	Test
Habitat	0.773	0.851	0.758–0.944	0.720	0.880	0.923	0.611	Test
Combined	0.853	0.912	0.845–0.979	0.820	0.920	0.953	0.719	Test

*AUROC* Area under the receiver operating characteristic curve, *CI* Confidence interval, *PPV* Positive predictive value, *NPV* Negative predictive value

In direct comparisons, the habitat model outperformed both the radiomics model (AUROCs: 0.843 and 0.814) and clinical model (AUROCs: 0.572 and 0.671) in the validation and test sets, respectively. These results highlight the superior capacity of habitat modeling to capture tumor heterogeneity compared to traditional radiomics. Notably, after feature selection, the combined model integrating clinical and habitat features achieved a higher AUROC than the habitat model on both the validation set (AUROC: 0.931) and the test set (AUROC: 0.912). According to evaluation metrics from various datasets, the combined model outperformed all other models. We also constructed a nomogram to facilitate clinical application (Fig. 4d).

**Fig. 5:**
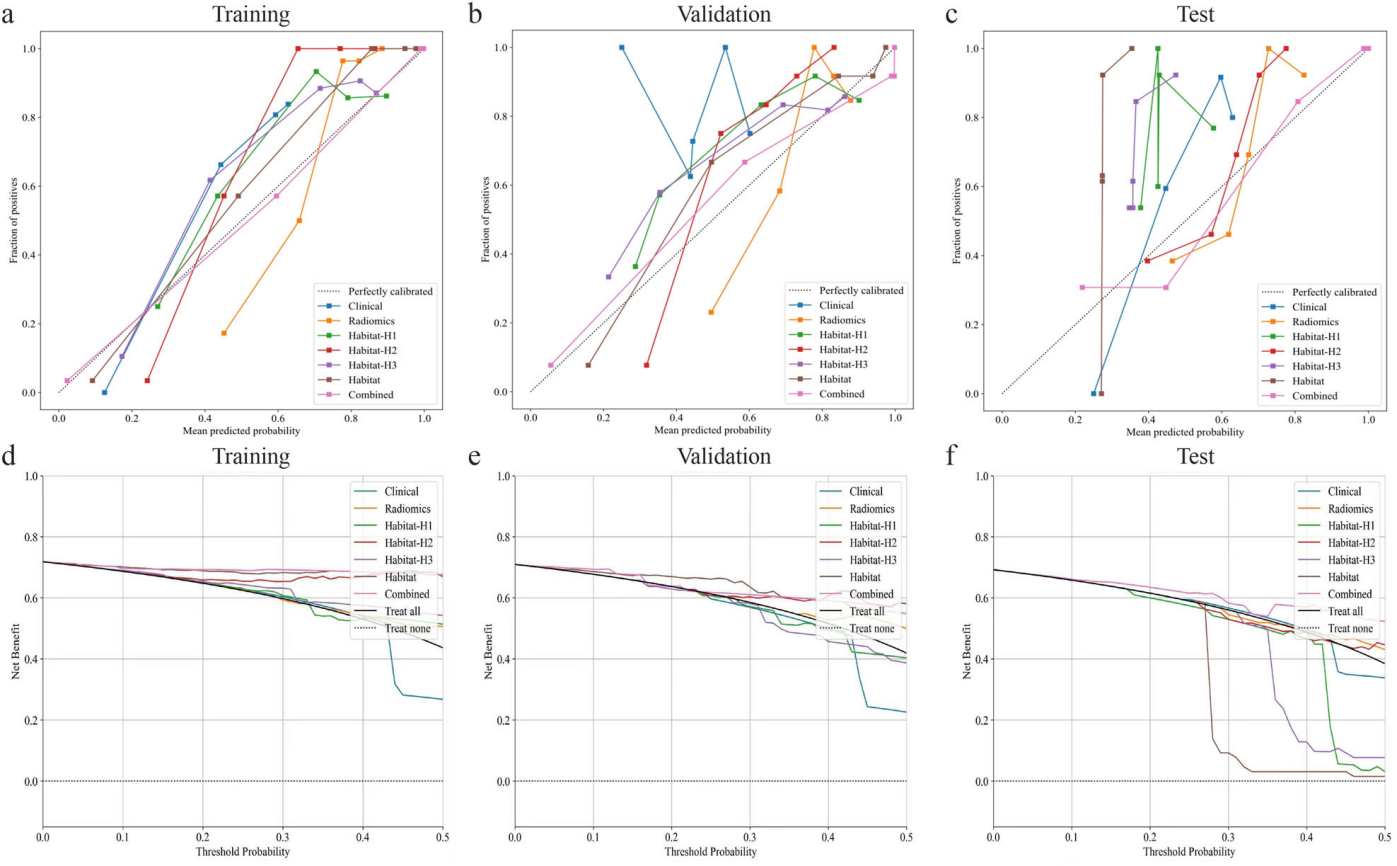
Calibration curves and decision curves for different models: calibration curves for training (**a**), validation (**b**), and test (**c**) sets. Decision curves for training (**d**), validation (**e**), and test (**f**) sets

Figure 5 displays the calibration and decision curves for the different models. The Hosmer–Lemeshow test demonstrated satisfactory calibration in all cohorts, as evidenced by nonsignificant Hosmer–Lemeshow test results in the training (0.899), validation (0.747), and test (0.178) sets, indicating a high level of concordance between predicted and actual outcomes and affirming the model’s reliability. DCA revealed that the combined model (nomogram) offered a substantial net benefit for probability estimation, outperforming other signatures in terms of net benefit. The key concepts and analytical methods of DCA are presented in Supplementary Material E3 [20, 21].

Decision curve analysis (Fig. 5) illustrated the net benefit of the models within the clinically relevant threshold probability range of 0.15–0.35. In the validation set, the habitat model showed good performance. The combined model yielded a modest net benefit over the treat-all strategy within a limited range of clinically plausible thresholds in the test set, whereas other models provided no consistent improvement over treat-all. The key concepts and analytical methods of DCA are presented in Supplementary Material E3 [20, 21].

**Fig. 6:**
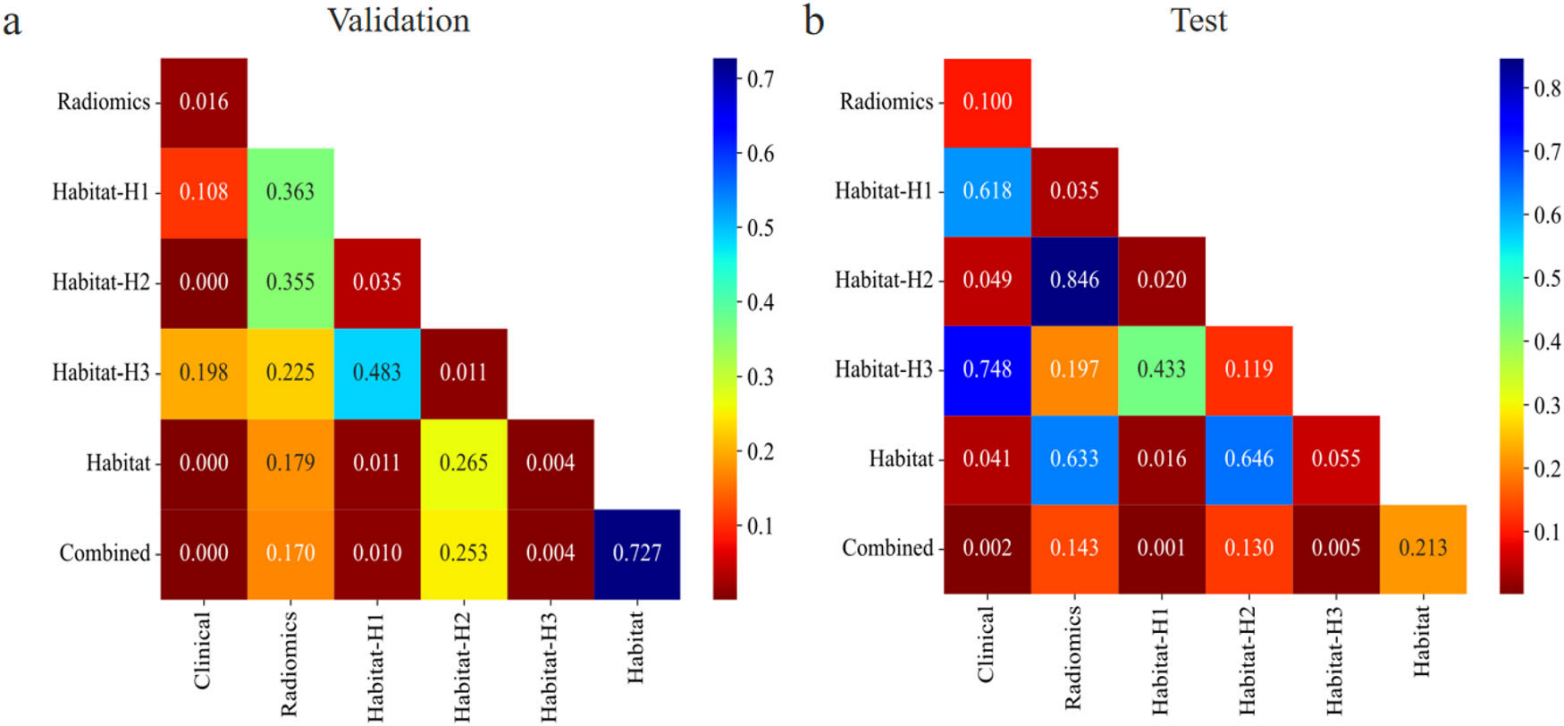
DeLong test for validation test (**a**) and test set (**b**)

Figure 6 presents the DeLong test results for different models on the validation and test sets. The combined model performed significantly better than the clinical, Habitat-H1 and Habitat-H3 models (*p* ≤ 0.010), while the habitat model demonstrated significant advantages in the validation and test sets, outperforming the clinical and Habitat-H1 models (*p* ≤ 0.041).

## Discussion

There is an urgent clinical need for preoperative, non-invasive differentiation between GB and SBM. In this study, we developed a tumor habitat model based on intratumoral heterogeneity. Compared with conventional radiomics and clinical models, the habitat model incorporating three subregions demonstrated superior performance in distinguishing GB from SBM. Notably, the Habitat-H2 subregion exhibited exceptional discriminative ability between the two tumor types. Furthermore, a combined model integrating habitat features with clinical variables (specifically, age) into a nomogram achieved optimal predictive performance and demonstrated good clinical net benefit.

As an advanced multi-shell technique, NODDI quantifies multidimensional microstructure: ICVF (neuron density), ISOVF (water distribution), and ODI (fiber complexity) [22]. Unlike conventional MRI, which is limited to morphology, or ADC, which lacks specificity due to multiple influencing factors [23], NODDI reveals specific microstructural differences between tumors and surrounding tissues. This capability provides a robust basis for the preoperative non-invasive differentiation of GB and SBM [24–26].

Habitat imaging captures local tumor heterogeneity [27–29], and in this study, the marginal enhancement subregion (Habitat-H2) yielded the best performance. In GB, this region is characterized by high ICVF due to dense cellular infiltration and proliferation. ODI increases significantly, reflecting the disorder of nerve fiber arrangement caused by tumor and glial infiltration, while ISOVF remains relatively low (isointense/slightly hyperintense) due to fewer necrotic components. In contrast, SBM margins exhibit lower ICVF and higher ISOVF; this is because although tumor cells exist, their density is lower than in GB, and frequent necrosis or liquefaction promotes isotropic water diffusion. Crucially, ODI in SBM remains unchanged or slightly elevated because the tumor exhibits expansile mass growth, causing limited interference with surrounding fibrous structures. These quantitative features align with the morphological differences—where GB’s irregular enhancement stems from abnormal vascular permeability and inflammatory-induced blood–brain barrier destruction [30], whereas SBM presents well-defined boundaries—allowing the habitat model to effectively distinguish the invasive nature of GB from the expansile growth of SBM.

Beyond the marginally enhancing Habitat-H2, the remaining subregions likely capture complementary microstructural milieus. Habitat-H1 in GB shows high ICVF, markedly high ISOVF, and heterogeneous ODI, suggesting hypercellularity with mixed structural organization and substantial free water. This heterogeneity likely reflects coexisting infiltrative strands, angiogenic remodeling, and patchy necrosis. In contrast, SBM displays mixed ICVF but consistently elevated ISOVF and predominantly high ODI, compatible with expansile metastatic nests containing prominent free water but less variable neurite disorganization. Habitat-H3 corresponds to the peritumoral solid region. In GB, it is characterized by low to near-normal ICVF, low ISOVF, and predominantly very high ODI, indicating non-enhancing infiltrative tissue or disorganized neurites with pronounced orientation dispersion. Conversely, SBM shows comparable ICVF and ISOVF levels with only moderately high ODI, reflecting perilesional tissue under vasogenic edema and pressure effects but without the pervasive microstructural disarray of GB. This stronger ODI elevation in GB explains Habitat-H3’s complementary discriminative value.

The DCA revealed that while the proposed habitat models are technically robust, their standalone clinical utility in the external test set was modest. Specifically, the habitat and radiomics models did not significantly outperform the baseline ‘treat-all’ strategy in the test cohort. This suggests that while intratumoral heterogeneity features are informative, they may need to be combined with clinical factors to achieve a tangible clinical benefit. This finding aligns with the need for cautious interpretation of radiomics models before clinical translation.

Habitat analysis and radiomics are often used together to address clinical challenges; however, the differences between these two methods require clarification. Habitat analysis partitions the tumor based on multimodal imaging to quantify the spatial distribution of distinct subregions, thereby emphasizing spatial heterogeneity and revealing complex microenvironmental structures [16, 17, 29]. In contrast, radiomics focuses on high-throughput feature extraction (morphology, texture, intensity) from a predefined ROI, such as the whole tumor, for machine learning-based classification [31, 32]. Our results demonstrate that the habitat model, which integrates features from three distinct subregions, outperforms whole-tumor radiomics. This advantage likely stems from the habitat approach’s ability to capture intrinsic spatial differences through data-driven clustering, rather than relying on artificially preset anatomical boundaries, ensuring a more effective representation of intratumoral heterogeneity.

Several limitations merit discussion. First, clinical implementation is constrained by the need for advanced hardware (3 T, high gradients) and prolonged acquisition times, which may limit accessibility and patient tolerance. Second, the complex post-processing pipeline currently relies on research software rather than integrated clinical workstations. Finally, a significant limitation is the lack of a direct comparison with expert neuroradiologist assessment. Without a head-to-head comparison in this specific cohort, we cannot quantify the incremental diagnostic value of our models over human interpretation. Consequently, our results should be interpreted primarily as a technical feasibility study, and future benchmarks against reader performance are necessary to establish clinical utility.

In conclusion, this study demonstrates the technical feasibility of NODDI-based habitat radiomics in characterizing the intratumoral heterogeneity of GB and SBM. While the habitat features alone showed limited clinical benefit in the external test set, their integration with clinical variables in a combined model improved predictive performance and net benefit. These findings suggest that habitat imaging may serve as a complementary tool rather than a standalone diagnostic solution.

## Supplementary information

**Additional file 1:** **E1**. Multi-center MRI Equipment and Image Processing. **E2**. Automatic segmentation. **E3**. The key concepts and analytical methods of decision curve analysis. **Figure E3**. Decision curves for the training (d), validation (e), and test (f) sets, with threshold probability = 0–1.0. **Table S1**. Sequence parameters of center A. **Table S2**. Sequence parameters of center B. **Table S3**. Performance of the habitat H1 models under different classifiers. **Table S4**. Performance of the habitat H2 models under different classifiers. **Table S5**. Performance of the habitat H3 models under different classifiers. **Table S6**. Performance of the habitat models under different classifiers. **Table S7**. Performance of the radiomics models under different classifiers. **Table S8**. Performance of the clinical models under different classifiers. **Fig S1**. Visualization of the feature selection process in the habitat H1 model. Coefficients of 10 fold cross validation (a), MSE of 10 fold cross validation (b), the histogram of the Rad-score based on the selected features (c). **Fig S2**. Visualization of the feature selection process in the habitat H2 model. Coefficients of 10 fold cross validation (a), MSE of 10 fold cross validation (b), the histogram of the Rad-score based on the selected features (c). **Fig S3**. Visualization of the feature selection process in the habitat H3 model. Coefficients of 10 fold cross validation (a), MSE of 10 fold cross validation (b), the histogram of the Rad-score based on the selected features (c). **Fig S4**. Visualization of the feature selection process in the habitat model. Coefficients of 10 fold cross validation (a), MSE of 10 fold cross validation (b), the histogram of the Rad-score based on the selected features (c). Eur Radiol Exp (2026) Luo W, Cheng Y, Pang C, et al. **Fig S5**. Visualization of the feature selection process in the radiomics model. Coefficients of 10 fold cross validation (a), MSE of 10 fold cross validation (b), the histogram of the Rad-score based on the selected features (c).

## Data Availability

The data could be obtained from the corresponding author upon reasonable request.

## Abbreviations

AUROC: Area under the receiver operating characteristic curve
GB: Glioblastoma
ICVF: Intracellular volume fraction
ISOVF: Isotropic volume fraction
NODDI: Neurite orientation dispersion and density imaging
ODI: Orientation dispersion index
ROI: Region of interest
SBM: Solitary brain metastasis
