# Supplementary material for: Cluster habitat-based diffusion MRI radiomics for differentiation of glioblastoma from solitary brain metastasis
Source: Eur Radiol Exp. 2026 Apr 27;10:56. doi: 10.1186/s41747-026-00717-y (PMC13121666; doi:10.1186/s41747-026-00717-y)
Supplement: Supplementary file 1 — Additional file 1: E1. Multi-center MRI Equipment and Image Processing. E2. Automatic segmentation. E3. The key concepts and analytical methods of decision curve analysis. Figure E3. Decision curves for the training (d), validation (e), and test (f) sets, with threshold probability = 0–1.0. Table S1. Sequence parameters of center A. Table S2. Sequence parameters of center B. Table S3. Performance of the habitat H1 models under different classifiers. Table S4. Performance of the habitat H2 models under different classifiers. Table S5. Performance of the habitat H3 models under different classifiers. Table S6. Performance of the habitat models under different classifiers. Table S7. Performance of the radiomics models under different classifiers. Table S8. Performance of the clinical models under different classifiers. Fig S1. Visualization of the feature selection process in the habitat H1 model. Coefficients of 10 fold cross validation (a), MSE of 10 fold cross validation (b), the histogram of the Rad-score based on the selected features (c). Fig S2. Visualization of the feature selection process in the habitat H2 model. Coefficients of 10 fold cross validation (a), MSE of 10 fold cross validation (b), the histogram of the Rad-score based on the selected features (c). Fig S3. Visualization of the feature selection process in the habitat H3 model. Coefficients of 10 fold cross validation (a), MSE of 10 fold cross validation (b), the histogram of the Rad-score based on the selected features (c). Fig S4. Visualization of the feature selection process in the habitat model. Coefficients of 10 fold cross validation (a), MSE of 10 fold cross validation (b), the histogram of the Rad-score based on the selected features (c). Eur Radiol Exp (2026) Luo W, Cheng Y, Pang C, et al. Fig S5. Visualization of the feature selection process in the radiomics model. Coefficients of 10 fold cross validation (a), MSE of 10 fold cross validation (b), the histogram of the Rad-score based on [file 41747_2026_717_MOESM1_ESM.pdf]

Cluster habitat-based diffusion MRI radiomics for differentiation of glioblastoma from solitary brain metastasis

ELECTRONIC SUPPLEMENTARY MATERIAL

Supplementary Material

E1. Multi-center MRI Equipment and Image Processing

Center A: MR acquisitions were performed using a 3.0-T MRI scanner (MAGNETOM Prisma; Siemens Healthcare, Erlangen, Germany) with a 64-channel head and neck integrated coil. MR data included conventional MRI sequences (axial T2-weighted image [T2WI], axial fluid-attenuated inversion recovery [FLAIR], axial T1-weighted image [T1WI], three-dimensional contrast-enhanced T1 magnetization prepared rapid gradient echo [CE-T1 MPRAGE]) and diffusion MRI. Diffusion MRI was performed using six different b-values (0, 500, 1000, 1500, 2000, and 2500 s/mm2) and every nonzero b-value in 30 encoding directions. CE-T1 MPRAGE was acquired after intravenous injection of 0.2 mL/kg gadopentetate dimeglumine (Magnevist, Bayer Schering Pharma AG, Berlin, Germany) using a high-pressure syringe, followed by a 20-mL saline flush at the same injection rate. CE-T1 MPRAGE images were obtained after contrast agent administration and reconstructed into 20 axial slices before use. NODDI parametric maps (including ICVF, ISOVF, and ODI) were calculated from the multi-b-value diffusion MRI data using in-house-developed post-processing software, NeuDiLab, based on the open-resource tool DIPY Toolbox (http://dipy.org). All MRI sequence parameters are listed in Supplementary Table 1.

Center B: MR acquisitions were performed using a 3.0-T MRI scanner (MAGNETOM Skyra; Siemens Healthcare, Erlangen, Germany) with a 32-channel head and neck integrated coil. Conventional MRI included axial T2WI, axial T1WI, axial T2-FLAIR, and 3D CE-T1WI, which was acquired after intravenous administration of 0.1 mmol/kg gadobutrol (Gadovist, Bayer AG, Berlin, Germany). For diffusion imaging, a diffusion spectrum imaging (DSI) scheme was implemented. In total, 128 diffusion-weighted samples were acquired, covering 16 distinct b-values: 200, 350, 400, 550, 750, 950, 1150, 1500, 1700, 1850, 1900, 2050, 2250, 2450, 2650, and 3000 s/mm^2. Diffusion data were further processed using NeuDiLab to generate NODDI parameter maps, including ICVF, ISOVF, and ODI. All MRI sequence parameters are listed in Supplementary Table 2.

E2. Automatic segmentation

All conventional MR images and NODDI parameter maps were registered to the FLAIR image using the open-source software ITK-SNAP (version 3.8.0, http://www.itksnap.org) to ensure that the ROI could be identified on all images. The ROI was defined as the solid tumor and was delineated by an automatic segmentation algorithm. Specifically, nnU-Net, which was trained on the BraTS 2020 challenge dataset, was used to automatically segment the lesions (1). The segmentations were then reviewed and revised by two radiologists (J.B. and X.M., with 5 and 10 years of experience, respectively), and the consensus segmentation was used as the ground truth.

1. Isensee F, Jaeger PF, Kohl SAA, et al. nnU-Net: a self-configuring method for deep learning-based biomedical image segmentation. Nat Methods 2021; 18(2): 203-211.

E3. The key concepts and analytical methods of decision curve analysis.

Decision curve analysis (DCA) was performed to evaluate the clinical utility of each model across clinically plausible threshold probabilities (pt). In this study, the management decision was defined as initiating a “GB-oriented management pathway” when the predicted probability of GB was ≥ pt; otherwise, an “SBM-oriented pathway” was chosen. For illustration, a GB-oriented pathway may involve prioritizing GB-oriented preoperative strategies, sampling plans, and auxiliary examinations. Net benefit was calculated as follows:

Net benefit = (TP/N) − (FP/N) × [pt/(1 − pt)]

where TP, FP, and N denote the numbers of true positives, false positives, and total patients, respectively. Two default strategies were included for reference:“Treat all” (initiating the GB-oriented pathway for all patients) and “Treat none” (initiating the GB-oriented pathway for none). Given the prevalence of GB in this study (approximately 196/279 ≈ 70%) and in alignment with the clinical team’s consensus, we restricted pt to 0.10–0.50 and defined 0.15–0.35 as the primary clinical threshold range. This reflects the lowest probability at which a GB-oriented pathway may offer an acceptable risk–benefit balance and indicates that above this upper bound, conventional assessment typically provides sufficient certainty.

Figure E3 presents the full view of the decision curves from Figure 5.

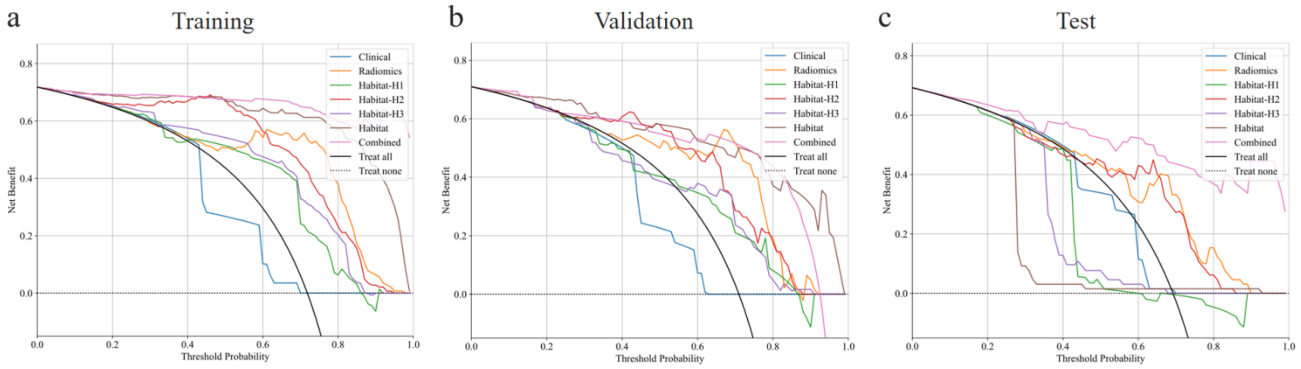

Figure E3. Decision curves for the training (d), validation (e), and test (f) sets, with threshold probability = 0–1.0.

Supplementary Tables

Table S1. Sequence parameters of center A.

Sequences	Slice orientation	TR/TE (ms)	Number of slices	Slice thickness	FOV (mm^2)	Scan time
T1WI	Axial	250/2.46	20	5.0 mm	220×220	38 s
T2WI	Axial	4090/99	20	5.0 mm	220×220	35 s
FLAIR	Axial	8000/81	20	5.0 mm	220×220	1 min 38 s
Multi-b-value diffusion MRI	Axial	2500/71	60	2.2 mm	220×220	6 min 34 s
CE-T1 MPRAGE	Sagittal	2300/2.32	176	0.9 mm	240×240	5 min 21 s

CE-T1 MPRAGE, contrast-enhanced T1 magnetization prepared rapid gradient echo.

Table S2. Sequence parameters of center B.

Sequences	Slice orientation	TR/TE (ms)	Number of slices	Slice thickness	FOV (mm^2)	Scan time
T1WI	Axial	1600/10	20	5.5 mm	230×230	1 min 46 s
T2WI	Axial	5500/117	20	5.5 mm	230×230	1 min 45 s
FLAIR	Axial	6000/81	20	5.5 mm	230×230	2 min 2 s
DSI	Axial	7000/107	60	3.0 mm	220×220	15 min 40 s
CE-T1WI	Axial	2300/2.32	192	0.9 mm	240×240	5 min 21 s

DSI, diffusion spectrum imaging.

Table S3. Performance of the habitat H1 models under different classifiers.

Model name	Accuracy	AUC	95% CI	Sensitivity	Specificity	PPV	NPV	Dataset
SVM	0.796	0.760	0.661 - 0.858	0.833	0.700	0.876	0.622	Training
SVM	0.774	0.757	0.614 - 0.899	0.795	0.722	0.875	0.591	Validation
SVM	0.600	0.619	0.465 - 0.772	0.500	0.800	0.833	0.444	Test
RandomForest	0.810	0.810	0.723 - 0.897	0.843	0.725	0.887	0.644	Training
RandomForest	0.710	0.705	0.563 - 0.846	0.705	0.722	0.861	0.500	Validation
RandomForest	0.640	0.629	0.520 - 0.797	0.580	0.760	0.829	0.475	Test
ExtraTrees	0.796	0.768	0.683 - 0.854	0.922	0.475	0.817	0.704	Training
ExtraTrees	0.548	0.660	0.515 - 0.805	0.455	0.778	0.833	0.368	Validation
ExtraTrees	0.520	0.611	0.467 - 0.755	0.420	0.720	0.750	0.383	Test
LightGBM	0.768	0.714	0.620 - 0.809	0.912	0.400	0.795	0.640	Training
LightGBM	0.613	0.628	0.482 - 0.773	0.614	0.611	0.794	0.393	Validation
LightGBM	0.520	0.568	0.426 - 0.709	0.400	0.760	0.769	0.388	Test

Table S4. Performance of the habitat H2 models under different classifiers.

Model name	Accuracy	AUC	95% CI	Sensitivity	Specificity	PPV	NPV	Dataset
SVM	0.873	0.919	0.872 - 0.966	0.863	0.900	0.957	0.720	Training
SVM	0.871	0.850	0.742 - 0.957	0.955	0.667	0.875	0.857	Validation
SVM	0.827	0.798	0.668 - 0.928	0.820	0.840	0.911	0.677	Test
RandomForest	0.908	0.967	0.942 - 0.992	0.882	0.975	0.989	0.765	Training
RandomForest	0.855	0.855	0.748 - 0.962	0.909	0.722	0.889	0.765	Validation
RandomForest	0.813	0.840	0.741 - 0.939	0.800	0.840	0.909	0.656	Test
ExtraTrees	0.972	0.989	0.974 - 1.000	0.961	1.000	1.000	0.909	Training
ExtraTrees	0.871	0.900	0.810 - 0.990	0.909	0.778	0.909	0.778	Validation
ExtraTrees	0.773	0.828	0.727 - 0.927	0.680	0.960	0.971	0.600	Test
LightGBM	0.944	0.967	0.941 - 0.993	0.980	0.850	0.943	0.944	Training
LightGBM	0.839	0.860	0.750 - 0.969	0.841	0.833	0.925	0.682	Validation
LightGBM	0.787	0.812	0.706 - 0.919	0.760	0.840	0.905	0.618	Test

Table S5. Performance of the habitat H3 models under different classifiers.

Model name	Accuracy	AUC	95% CI	Sensitivity	Specificity	PPV	NPV	Dataset
SVM	0.831	0.814	0.725 - 0.903	0.873	0.725	0.890	0.690	Training
SVM	0.710	0.723	0.574 - 0.872	0.682	0.778	0.882	0.500	Validation
SVM	0.680	0.701	0.571 - 0.831	0.620	0.800	0.861	0.513	Test
RandomForest	0.831	0.828	0.744 - 0.913	0.863	0.750	0.898	0.682	Training
RandomForest	0.758	0.708	0.550 - 0.866	0.795	0.667	0.854	0.571	Validation
RandomForest	0.720	0.638	0.486 - 0.790	0.880	0.400	0.746	0.625	Test
ExtraTrees	0.662	0.779	0.691 - 0.867	0.608	0.800	0.886	0.444	Training
ExtraTrees	0.613	0.688	0.531 - 0.844	0.545	0.778	0.857	0.412	Validation
ExtraTrees	0.653	0.704	0.574 - 0.834	0.600	0.760	0.833	0.487	Test
LightGBM	0.810	0.830	0.746 - 0.914	0.853	0.700	0.879	0.651	Training
LightGBM	0.742	0.679	0.516 - 0.841	0.795	0.611	0.833	0.550	Validation
LightGBM	0.693	0.676	0.530 - 0.821	0.740	0.600	0.787	0.536	Test

Table S6. Performance of the habitat models under different classifiers.

Model name	Accuracy	AUC	95% CI	Sensitivity	Specificity	PPV	NPV	Dataset
SVM	0.965	0.989	0.977 - 1.000	0.971	0.950	0.980	0.927	Training
SVM	0.871	0.929	0.862 - 0.995	0.886	0.833	0.929	0.750	Validation
SVM	0.733	0.851	0.758 - 0.944	0.720	0.880	0.923	0.611	Test
RandomForest	0.908	0.967	0.944 - 0.991	0.873	1.000	1.000	0.755	Training
RandomForest	0.855	0.865	0.755 - 0.974	0.864	0.833	0.927	0.714	Validation
RandomForest	0.693	0.770	0.646 - 0.894	0.620	0.840	0.886	0.525	Test
ExtraTrees	0.859	0.929	0.888 - 0.970	0.843	0.900	0.956	0.692	Training
ExtraTrees	0.871	0.880	0.781 - 0.979	0.864	0.889	0.950	0.727	Validation
ExtraTrees	0.800	0.792	0.665 - 0.918	0.840	0.720	0.857	0.692	Test
LightGBM	0.923	0.986	0.972 - 1.000	0.892	1.000	1.000	0.784	Training
LightGBM	0.887	0.876	0.765 - 0.988	0.909	0.833	0.930	0.789	Validation
LightGBM	0.653	0.777	0.655 - 0.900	0.500	0.960	0.962	0.490	Test

Table S7. Performance of the radiomics models under different classifiers.

Model name	Accuracy	AUC	95% CI	Sensitivity	Specificity	PPV	NPV	Dataset
SVM	0.880	0.926	0.879 - 0.972	0.873	0.900	0.957	0.735	Training
SVM	0.903	0.843	0.703 - 0.983	0.932	0.833	0.932	0.833	Validation
SVM	0.773	0.814	0.705 - 0.923	0.700	0.920	0.946	0.605	Test
RandomForest	0.901	0.952	0.919 - 0.985	0.912	0.875	0.949	0.795	Training
RandomForest	0.839	0.835	0.708 - 0.961	0.864	0.778	0.905	0.700	Validation
RandomForest	0.747	0.785	0.665 - 0.905	0.660	0.920	0.943	0.575	Test
ExtraTrees	0.824	0.919	0.871 - 0.968	0.775	0.950	0.975	0.623	Training
ExtraTrees	0.839	0.842	0.718 - 0.965	0.864	0.778	0.905	0.700	Validation
ExtraTrees	0.760	0.804	0.699 - 0.908	0.680	0.920	0.944	0.590	Test
LightGBM	0.810	0.894	0.843 - 0.946	0.775	0.900	0.952	0.610	Training
LightGBM	0.839	0.836	0.711 - 0.961	0.864	0.778	0.905	0.700	Validation
LightGBM	0.827	0.819	0.697 - 0.941	0.860	0.760	0.878	0.731	Test

Table S8. Performance of the clinical models under different classifiers.

Model name	Accuracy	AUC	95% CI	Sensitivity	Specificity	PPV	NPV	Dataset
SVM	0.549	0.626	0.533 - 0.718	0.471	0.750	0.828	0.357	Training
SVM	0.532	0.572	0.420 - 0.723	0.477	0.667	0.778	0.343	Validation
SVM	0.653	0.671	0.531 - 0.809	0.580	0.800	0.853	0.488	Test
RandomForest	0.664	0.649	0.554 - 0.743	0.958	0.313	0.765	0.761	Training
RandomForest	0.547	0.584	0.486 - 0.681	0.958	0.254	0.750	0.722	Validation
RandomForest	0.667	0.583	0.434 - 0.734	0.800	0.400	0.727	0.500	Test
ExtraTrees	0.576	0.681	0.593 - 0.769	0.453	0.862	0.885	0.407	Training
ExtraTrees	0.639	0.606	0.457 - 0.755	0.862	0.454	0.785	0.588	Validation
ExtraTrees	0.720	0.606	0.457 - 0.755	0.860	0.440	0.754	0.611	Test
LightGBM	0.564	0.568	0.529 - 0.608	0.092	1.000	1.000	0.320	Training
LightGBM	0.605	0.539	0.460 - 0.617	0.705	0.375	0.724	0.352	Validation
LightGBM	0.640	0.574	0.456 - 0.691	0.780	0.360	0.709	0.450	Test

Supplementary Figure

Figure S1

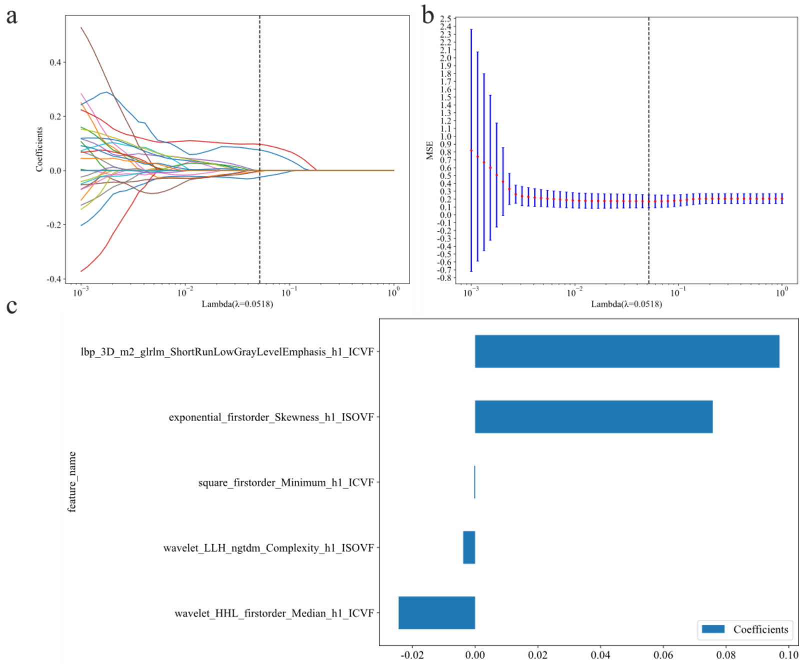

Fig S1. Visualization of the feature selection process in the habitat H1 model. Coefficients of 10 fold cross validation (a), MSE of 10 fold cross validation (b), the histogram of the Rad-score based on the selected features (c).

Figure S2

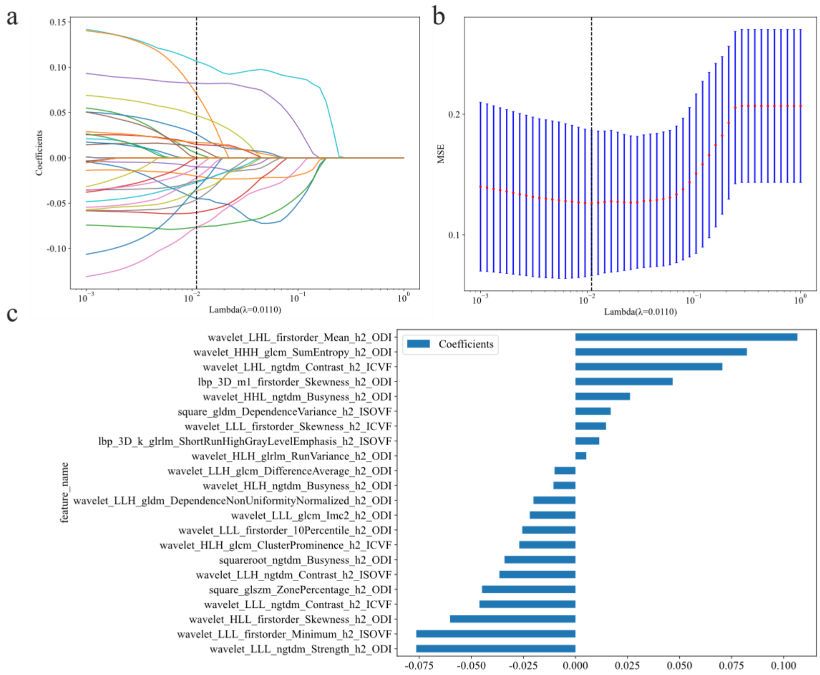

Fig S2. Visualization of the feature selection process in the habitat H2 model. Coefficients of 10 fold cross validation (a), MSE of 10 fold cross validation (b), the histogram of the Rad-score based on the selected features (c).

Figure S3

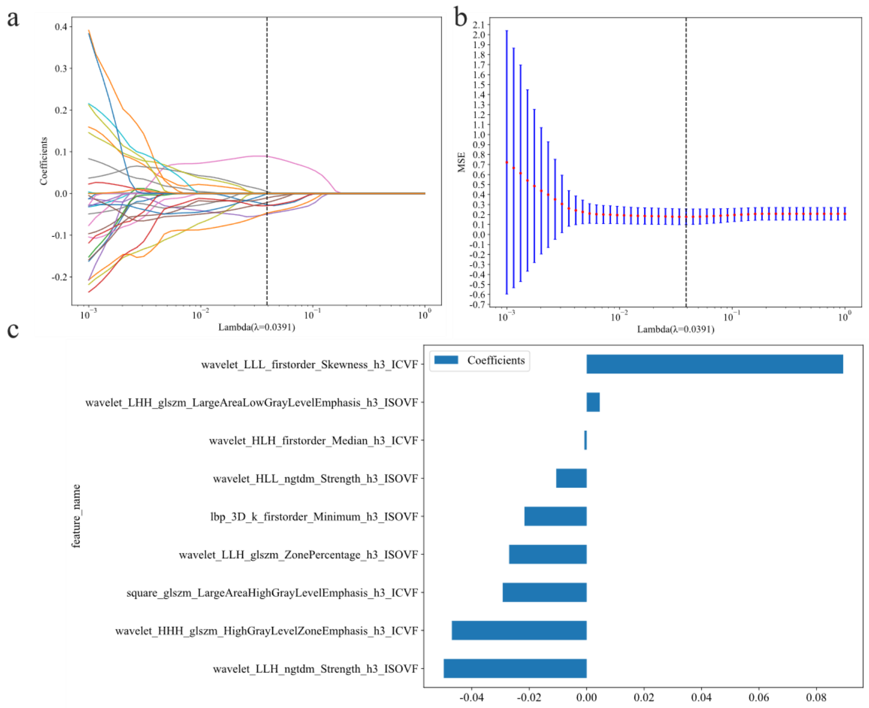

Fig S3. Visualization of the feature selection process in the habitat H3 model. Coefficients of 10 fold cross validation (a), MSE of 10 fold cross validation (b), the histogram of the Rad-score based on the selected features (c).

Figure S4

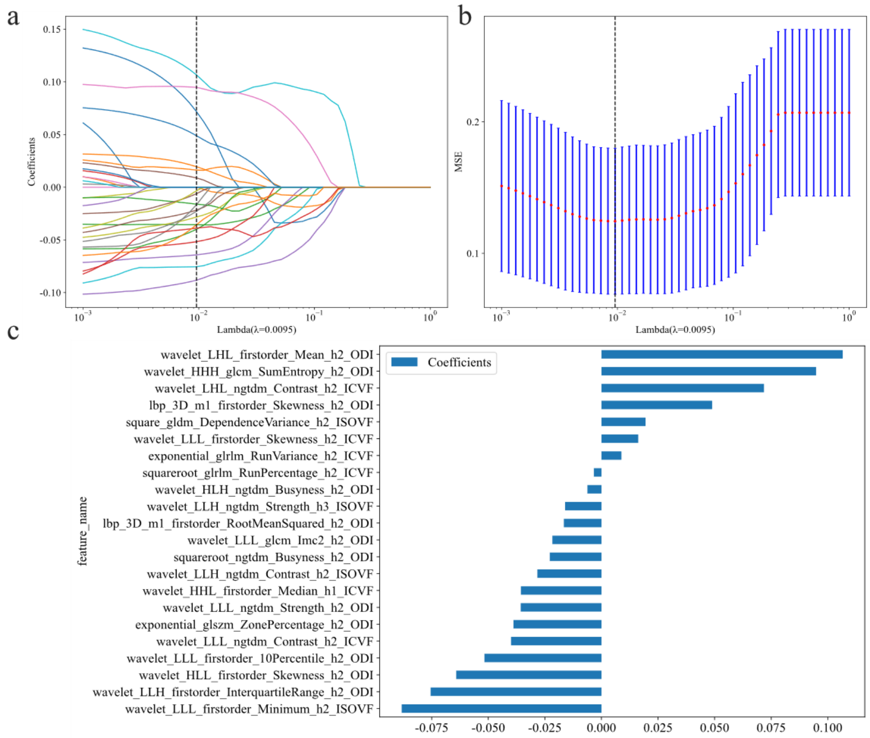

Fig S4. Visualization of the feature selection process in the habitat model. Coefficients of 10 fold cross validation (a), MSE of 10 fold cross validation (b), the histogram of the Rad-score based on the selected features (c).

Figure S5

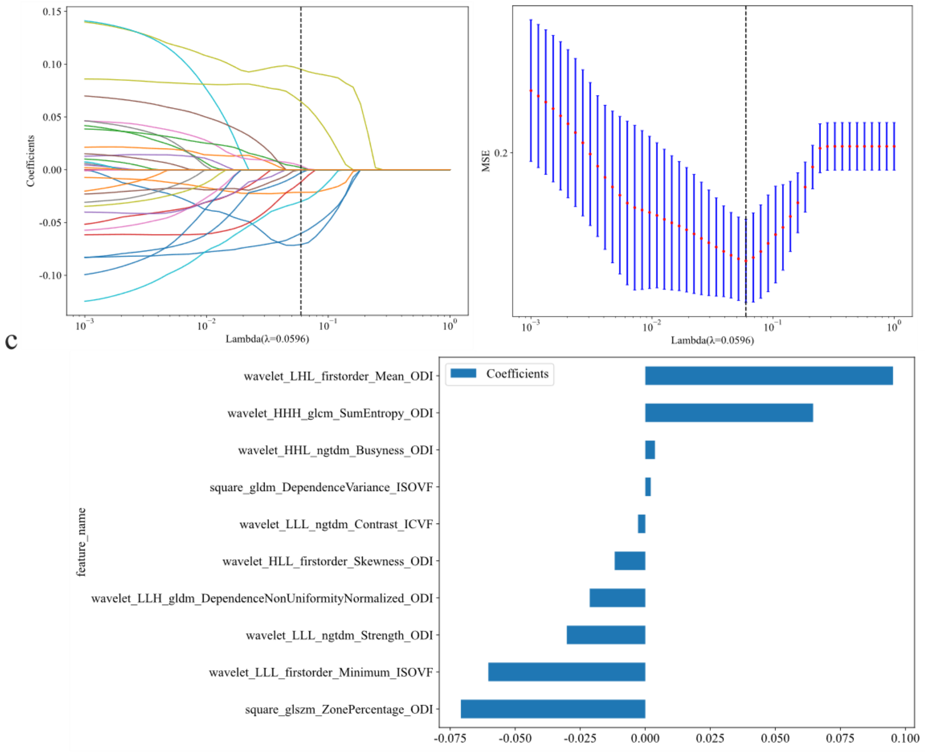

Fig S5. Visualization of the feature selection process in the radiomics model. Coefficients of 10 fold cross validation (a), MSE of 10 fold cross validation (b), the histogram of the Rad-score based on the selected features (c).
